# Nutritional support in severe traumatic brain injury

**DOI:** 10.1186/cc9795

**Published:** 2011-03-11

**Authors:** MN Cote, F Lauzier, V Bibeau, P Labbe, AF Turgeon

**Affiliations:** 1 CHA-Hôpital de l'Enfant-Jésus, Université Laval, Quebec, Canada

## Introduction

Clinical guidelines recommend full caloric replacement within 7 days after severe traumatic brain injury (TBI) since it may improve clinical outcomes. However, enteral feeding is often poorly tolerated in this population. We hypothesized that most patients with severe TBI do not receive adequate caloric and protein intake.

## Methods

We performed a retrospective cohort study of randomly selected patients with severe TBI (GCS ≤8) identified with ICD-10 codes and admitted to a 24-bed ICU of a Canadian level 1 trauma center between January 2005 and December 2006. We excluded patients <16 years old, with penetrating TBI or mechanically ventilated for <48 hours. Using a standardized pretested case report form, we collected daily kilocalories and proteins (ordered and received), sedation, use of prokinetic drugs and post-pyloric access. The primary endpoint was achievement of ≥90% of caloric and protein requirement within 7 days. Secondary endpoints were factors associated with achievement of nutritional goals and with gastric intolerance (one episode of residuals ≥250 ml/4 hours). A sample size of 100 patients was required to obtain a margin of error of 9%. Student *t *and chi-square tests were used to compare continuous data and proportions. We obtained ethics approval.

## Results

Among the 109 patients included, 82.6% were men (mean age 40.5 ± 20.5 years, GCS 3.7 ± 1.3 and BMI 25.3 ± 5.1 kg/m^2^). Patients had 1,204 potential feeding days. Ninety-six patients (88.1%) were fed by day 3. Mean caloric and protein orders were 32.6 ± 4.8 kcal/kg and 1.4 ± 0.2 g/kg, respectively. Two patients never received enteral nutrition. Nutrition was started at a mean rate of 32.6 ± 9.3% of the nutritional goal using the stomach as the initial access in 97.2%. The achievement of caloric, protein and both requirements was successful in 48 (44.0%, 95% CI = 34.7 to 53.4%), 64 (58.7%, 95% CI = 49.5 to 68.0%) and 42 (38.5, 95% CI = 29.4 to 47.7%) patients during the first week. The most associated factor with unsuccessful nutrition was gastric intolerance (RR = 1.40. 95% CI = 1.11 to 1.88, *P *< 0.01), which occurred in 49.5% patients. Factors associated with gastric intolerance were young age (*P *< 0.001), increased intracranial pressure (*P *< 0.001), high opioid doses (*P *= 0.004) and nonuse of prokinetic drugs (*P *= 0.05).

## Conclusions

Most patients with severe TBI did not achieve nutritional goals within 7 days, partially due to high gastric residuals. Although we identified factors associated high gastric residuals, improving feeding tolerance is unlikely to be the only intervention to significantly improve nutritional intakes.

